# Three-party quantum private computation of cardinalities of set intersection and union based on GHZ states

**DOI:** 10.1038/s41598-020-77579-w

**Published:** 2020-12-17

**Authors:** Cai Zhang, Yinxiang Long, Zhiwei Sun, Qin Li, Qiong Huang

**Affiliations:** 1 College of Mathematics and Informatics, South China Agricultural University, Guangzhou, 510642 China; 2 Department of Automation Engineering, Guangdong Technical College of Water Resources and Electric Engineering, Guangzhou, 510925 China; 3 School of Artificial Intelligence, Shenzhen PolyTechnic, Shenzhen, 518055 China; 4 School of Computer Science, Xiangtan University, Xiangtan, 411105 China

**Keywords:** Quantum information, Quantum mechanics

## Abstract

Private Set Intersection Cardinality (PSI-CA) and Private Set Union Cardinality (PSU-CA) are two cryptographic primitives whereby two or more parties are able to obtain the cardinalities of the intersection and the union of their respective private sets, and the privacy of their sets is preserved. In this paper, we propose a three-party protocol to finish these tasks by using quantum resources, where every two, as well as three, parties can obtain the cardinalities of the intersection and the union of their private sets with the help of a semi-honest third party (TP). In our protocol, GHZ states play a role in encoding private information that will be used by TP to compute the cardinalities. We show that the presented protocol is secure against well-known quantum attacks. In addition, we analyze the influence of six typical kinds of Markovian noise on our protocol.

## Introduction

Quantum key distribution is one kind of important cryptographic protocols based on quantum mechanics, in which any outside eavesdropper attempting to obtain the secret key shared by two users will be detected. The successful detection comes from Heisenberg’s uncertainty principle: the measurement of a quantum system, which is required to obtain information of that system, will generally disturb it. The disturbances provide two users with the information that there exists an outside eavesdropper, and they can therefore abort the communication. Nowadays, most people need to share some of their private information for certain services such as products recommendation for online shopping and collaborations between two companies depending on their comm interests. Private Set Intersection Cardinality (PSI-CA) and Private Set Union Cardinality (PSU-CA), which are two primitives in cryptography, involve two or more users who intend to obtain the cardinalities of the intersection and the union of their private sets through the minimum information disclosure of their sets^1–3^.

The definition of Private Set Intersection (PSI), also called Private Matching (PM), was proposed by Freedman^4^. They employed balanced hashing and homomorphic encryption to design two PSI protocols and also investigated some variants of PSI. In 2012, Cristofaro et al.^1^ developed several PSI-CA and PSU-CA protocols with linear computation and communication complexity based on the Diffie-Hellman key exchange which blinds the private information. Their protocols were the most efficient compared with the previous classical related ones. There are also other classical PSI-CA or PSU-CA protocols^5–8^. Nevertheless, the security of these protocols relies on the unproven difficulty assumptions, such as discrete logarithm, factoring, and quadratic residues assumptions, which will be insecure when quantum computers are available^9–11^.

For the sake of improving the security of PSI-CA protocols for two parties, Shi et al.^3^ designed a probabilistic protocol where multi-qubit entangled states, complicated oracle operators, and measurements in high *N*-dimensional Hilbert space were utilized. And the same method in Ref.^3^ was later used to develop a PSI-CA protocol for multiple parties^12^. For easy implementation of a protocol, Shi et al.^13^ leveraged Bell states to construct another protocol for PSI-CA and PSU-CA problems that was more practical than that in Ref.^3^. In both protocols Ref.^3^ and Ref.^13^, only two parties who intend to get the cardinalities of the intersection and the union of their private sets are involved. Although Ref.^12^ works for multiple parties, it only solves the PSI-CA problem and requires multi-qubit entangled states, complicated oracle operators, and measurements. It then interests us that how we could design a more practical protocol for multiple parties to simultaneously solve PSI-CA and PSU-CA problems. Inspired by Shi et al.’s work, we are thus trying to design a three-party protocol to solve PSI-CA and PSU-CA problems, where every two and three parties can obtain the cardinalities of the intersection and the union of their respective private sets with the aid of a semi-honest third party (TP). TP is semi-honest means that he loyally executes the protocol, makes a note of all the intermediate results, and might desire to take other parties’ private information, but he cannot collude with dishonest parties. We then give a detailed analysis of the presented protocol’s security. Besides, the influence of six typical kinds of Markovian noise on our protocol is also analyzed.

## Preliminaries

First of all, we introduce the properties of GHZ states, and then give a detailed description of our protocol.

The standard GHZ three-qubit state is usually given by

1 $$\begin{aligned} |\varphi _{000}\rangle =\frac{1}{\sqrt{2}}(|000\rangle +|111\rangle ). \end{aligned}$$

Let $U=ZX$, where $X=|0\rangle \langle 1|+|1\rangle \langle 0|$ and $Z=|0\rangle \langle 0|-|1\rangle \langle 1|$. Combining *U* and $I=|0\rangle \langle 0|+|1\rangle \langle 1|$, we can deduce the following equations:

2 $$\begin{gathered} (U \otimes I \otimes I)|\varphi _{{000}} \rangle = \frac{1}{{\sqrt 2 }}(|011\rangle - |100\rangle ) = |\varphi _{{100}} \rangle , \hfill \\ (I \otimes U \otimes I)|\varphi _{{000}} \rangle = \frac{1}{{\sqrt 2 }}(|101\rangle - |010\rangle ) = |\varphi _{{010}} \rangle , \hfill \\ (I \otimes I \otimes U)|\varphi _{{000}} \rangle = \frac{1}{{\sqrt 2 }}(|110\rangle - |001\rangle ) = |\varphi _{{001}} \rangle , \hfill \\ (U \otimes U \otimes I)|\varphi _{{000}} \rangle = \frac{1}{{\sqrt 2 }}(|001\rangle + |110\rangle ) = |\varphi _{{110}} \rangle , \hfill \\ (U \otimes I \otimes U)|\varphi _{{000}} \rangle = \frac{1}{{\sqrt 2 }}(|101\rangle + |010\rangle ) = |\varphi _{{101}} \rangle , \hfill \\ (I \otimes U \otimes U)|\varphi _{{000}} \rangle = \frac{1}{{\sqrt 2 }}(|011\rangle + |100\rangle ) = |\varphi _{{011}} \rangle , \hfill \\ (U \otimes U \otimes U)|\varphi _{{000}} \rangle = \frac{1}{{\sqrt 2 }}(|000\rangle - |111\rangle ) = |\varphi _{{111}} \rangle . \hfill \\ \end{gathered}$$

Note that Eqs. (1)–(2) form a basis (we call it GHZ basis hereafter) for the space of the three-qubit quantum system.

## Results

### The proposed protocol

Our protocol will satisfy the following requirements:

1. *Correctness* The respective cardinalities of the intersection and the union of every two and three parties’ sets are correct.
2. *Privacy* TP and dishonest parties cannot learn about the elements of any party’s set.
3. *Fairness* All the parties are perfect peer entities and they can get the cardinalities with equal opportunities.

In our protocol, TP is assumed to be semi-honest which means that he honestly follows the protocol, writes down all the intermediate results and might attempt to obtain the elements of any party’s private set, but he cannot be collusive with any dishonest party.

Suppose that Alice, Bob and Charlie have private sets $A=\{a_{1}, a_{2}, \ldots , a_{l} \}$, $B=\{b_{1}, b_{2}, \ldots ,b_{m} \}$ and $C=\{c_{1}, c_{2}, \ldots ,c_{n} \}$, respectively, and each element of these sets lies in $Z_{p}$, where $Z_{p}=\{0,1,2 \ldots , p-1\}$ and *p* is a large prime number. TP helps compute the cardinalities $|A\cap B|$ ($|A\cup B|$), $|A\cap C|$ ($|A\cup C|$), $|B\cap C|$ ($|B\cup C|$), and $|A\cap B\cap C|$ ($|A\cup B \cup C|$). Our protocol works as follows:

- (*Step 1*) Alice, Bob and Charlie run a Quantum Key Agreement (QKA) protocol^14–16^ to share a secret non-zero binary key *k* that corresponds to a secret integer over $Z_{p}$. Then, Alice, Bob and Charlie compute
  $$\begin{aligned} A^{*}=\left\{ k a_{1}\ (\bmod \ p), \ldots , k a_{l}\ (\bmod \ p)\right\} , \\ B^{*}=\left\{ k b_{1}\ (\bmod \ p), \ldots , k b_{m}\ (\bmod \ p)\right\} , \end{aligned}$$
  and
  $$\begin{aligned} C^{*}=\left\{ k c_{1}\ (\bmod \ p), \ldots , k c_{n}\ (\bmod \ p)\right\} , \end{aligned}$$
  respectively.

- (*Step 2*) Alice, Bob and Charlie encode their respective sets $A^{*}$, $B^{*}$ and $C^{*}$ into three private vectors over $Z_{p}^{2}$ as follows: Alice constructs a private vector $(x_{0},x_{1},\ldots ,x_{p-1})\in Z_{2}^{p}$, where $x_{i}=1$ if $i \in A^{*}$ and $x_{i}=0$, otherwise, for $i=0,1,\ldots ,p-1$; Bob produces a private vector $(y_{0},y_{1},\ldots ,y_{p-1})\in Z_{2}^{p}$, where $y_{i}=1$ if $i \in B^{*}$ and $y_{i}=0$, otherwise, for $i=0,1,\ldots ,p-1$; Charlie generates a private vector $(z_{0},z_{1},\ldots ,z_{p-1})\in Z_{2}^{p}$, where $z_{i}=1$ if $i \in C^{*}$ and $z_{i}=0$, otherwise, for $i=0,1,\ldots ,p-1$.

- (*Step 3*) TP prepares *p* GHZ states ($G_{A0},G_{B0},G_{C0}$), ($G_{A1},G_{B1},G_{C1}$), $\ldots$, ($G_{A(p-1)},G_{B(p-1)},G_{C(p-1)}$), with each GHZ state being in the state $|\varphi _{000}\rangle$. These GHZ states are referred to as encoding states. Next, TP divides all particles into three ordered sequences: $(G_{A0},G_{A1},\ldots ,$ $G_{A(p-1)})$, $(G_{B0},G_{B1},\ldots ,G_{B(p-1)})$, and $(G_{C0},G_{C1},\ldots ,$ $G_{C(p-1)})$, which are denoted as $T_{A}$, $T_B$, and $T_C$, respectively.

- (*Step 4*) TP generates 3*d* decoy particles, each of which is randomly chosen from the set $\{|0\rangle ,|1\rangle ,|+\rangle ,|-\rangle \}$, where $|+\rangle =\frac{1}{\sqrt{2}}(|0\rangle +|1\rangle )$ and $|-\rangle =\frac{1}{\sqrt{2}}(|0\rangle -|1\rangle )$. Afterwards, TP randomly inserts *d* decoy particles into $T_{A}$ ($T_{B}$, $T_{C}$) to form a new sequence $T_{A}^{\prime }$ ($T_{B}^{\prime }$, $T_{C}^{\prime }$). TP then sends $T_{A}^{\prime }$ ($T_{B}^{\prime }$, $T_{C}^{\prime }$) to Alice (Bob, Charlie) through a quantum channel.

- (*Step 5*) Confirming that Alice (Bob, Charlie) has successfully received $T_{A}^{\prime }$ ($T_{B}^{\prime }$, $T_{C}^{\prime }$), TP announces the inserted positions of all the *d* decoy particles in $T_{A}^{\prime }$ ($T_{B}^{\prime }$, $T_{C}^{\prime }$) and their corresponding measurement bases. Then, Alice (Bob, Charlie) measures all the decoy particles in the correct bases and announces the measurement results to TP. Next, TP compares the measurement results with their corresponding initial states. If the error rate is higher than the threshold determined by the channel noise, this protocol will be aborted. Otherwise, the protocol will continue to the next step.

- (*Step 6*) Alice (Bob, Charlie) removes all the decoy particles from the sequence $$T_{A}^{\prime }$$ ($$T_{B}^{\prime }$$, $$T_{C}^{\prime }$$) to obtain the initial sequence $$T_{A}$$ ($$T_{B}$$, $$T_{C}$$). For each particle in $$T_{A}$$ ($$T_{B}$$, $$T_{C}$$), Alice (Bob, Charlie) performs *U* on $$G_{Ai}$$ ($$G_{Bi}$$, $$G_{Ci}$$) ($$i = 0, 1, \ldots , P-1$$) if $$x_i = 
1$$ ($$y_i = 1,z_i = 1$$); otherwise, Alice (Bob, Charlie) does nothing on $$G_{Ai}$$ ($$G_{Bi}$$, $$G_{Ci}$$).

- (*Step 7*) Alice (Bob, Charlie) prepares *d* decoy particles to detect eavesdropping. Each decoy states is randomly chosen from the set $\{|0\rangle ,|1\rangle ,$ $|+\rangle ,|-\rangle \}$. Later, Alice (Bob, Charlie) randomly inserts these *d* decoy particles into $T_{A}$ ($T_{B}, T_{C}$) to form a new sequence $T_{A}^{*}$ ($T_{B}^{*}, T_{C}^{*}$), and writes down the positions and the states of these inserted states. At last, Alice (Bob, Charlie) sends $T_{A}^{*}$ ($T_{B}^{*}, T_{C}^{*}$) to TP through a quantum channel.

- (*Step 8*) Confirming that TP has successfully received $T_{A}^{*}$ ($T_{B}^{*}$, $T_{C}^{*}$), Alice (Bob, Charlie) announces the inserted positions of all *d* decoy particles in $T_{A}^{*}$ ($T_{B}^{*}$, $T_{C}^{*}$) and their corresponding measurement bases. TP measures all decoy particles in the correct bases and announces the measurement results. Alice (Bob, Charlie) then compares the measurement results with their corresponding initial states. If the error rate is higher than the threshold determined by the channel noise, the protocol will be aborted. Otherwise, the protocol will continue to the next step.

- (*Step 9*) TP discards all decoy particles from $T_{A}^{*}$ ($T_{B}^{*}$, $T_{C}^{*}$) to attain $T_{A}$ ($T_{B}$, $T_{C}$). TP then selects eight variables $S_{000}$, $S_{100}$, $S_{010}$, $S_{001}$, $S_{110}$, $S_{101}$, $S_{011}$ and $S_{111}$ as the counters and sets them all to zero. Next, TP measures each trio ($G_{Ai}G_{Bi}G_{Ci}$) ($i=0,1, \ldots ,p-1$) in the GHZ basis. If the measurement result is $|\varphi _{r}\rangle$ ($r\in \{0,1\}^{3}$), TP computes $S_{r}=S_{r}+1$. Finally, TP can calculate the cardinalities $|A\cap B|=S_{110}+S_{111}$, $|A\cap C|=S_{101}+S_{111}$, $|B\cap C|=S_{011}+S_{111}$, $|A\cap B \cap C|=S_{111}$, $|A\cup B|=p-S_{000}-S_{001}$, $|A\cup C|=p-S_{000}-S_{010}$, $|B\cup C|=p-S_{000}-S_{100}$, and $|A \cup B\cup C|=p-S_{000}$.

### Correctness and security analyzes

In this section, we will analyze the correctness and the security of our protocol. Let us first give the analysis of the correctness.

#### Correctness

On the one hand, for any $x,y \in Z_{p}$ and $k \in Z_{P}-\{0\}$ , $x=y$ if and only if $kx=ky \ (mod\ p)$. It is easy to deduce that

3 $$\begin{aligned} \begin{aligned} |A\cap B|&=|A^{*}\cap B^{*}|,\\ |A\cap C|&=|A^{*}\cap C^{*}|,\\ |B\cap C|&=|B^{*}\cap C^{*}|,\\ |A\cap B \cap C|&=|A^{*}\cap B^{*} \cap C^{*}|,\\ |A\cup B|&=|A^{*}\cup B^{*}|,\\ |A\cup C|&=|A^{*}\cup C^{*}|,\\ |B\cup C|&=|B^{*}\cup C^{*}|, \\ |A \cup B\cup C|&=|A^{*} \cup B^{*}\cup C^{*}|. \end{aligned} \end{aligned}$$

On the other hand, by the coding rules in (Step 2) and (Step 6), for any $i\in Z_p$, if $i\notin A^{*}\wedge i\notin B^{*} \wedge i\notin C^{*}$ ($i\in \overline{A^{*}\cup B^{*} \cup C^{*}}$), then $x_{i}=y_{i}=z_{i}=0$, and Alice (Bob, Charlie) does nothing on the particle $G_{Ai}$ ($G_{Bi}$,$G_{Ci}$) when she (he, he) receives it. TP will get the measurement result $|\varphi _{000}\rangle$ in step 9. Clearly, $S_{000}$ is used to count the number of GHZ trios whose states are the same as their original states. The cardinality of the union of the sets $A^{*}$, $B^{*}$ and $C^{*}$ therefore equals $p-S_{000}$. Namely, $|A\cup B \cup C|= |A^{*}\cup B^{*} \cup C^{*}|= p-S_{000}$. Similarly, we can analyze other cases where *i* belonging to different sets corresponds to three parties’ different operations; see Table 1. From step 9, we have

4 $$\begin{aligned} S_{100}= & {} |A^{*} \cap {\overline{B^{*}}} \cap {\overline{C^{*}}}|,\nonumber \\ S_{010}= & {} |{\overline{A^{*}}} \cap B^{*} \cap {\overline{C^{*}}}|,\nonumber \\ S_{001}= & {} |{\overline{A^{*}}} \cap {\overline{B^{*}}} \cap C^{*}|,\nonumber \\ S_{110}= & {} |A^{*} \cap B^{*} \cap {\overline{C^{*}}}|,\nonumber \\ S_{101}= & {} |A^{*} \cap {\overline{B^{*}}} \cap C^{*}|,\nonumber \\ S_{011}= & {} |{\overline{A^{*}}} \cap B^{*}\cap C^{*}|,\nonumber \\ S_{111}= & {} |A^{*} \cap B^{*}\cap C^{*}|, \end{aligned}$$

where $${\overline{A^{*}}}=Z_{p}-A^{*}$$, $${\overline{B^{*}}}=Z_{p}-B^{*}$$, and $${\overline{C^{*}}}=Z_{p}-C^{*}$$.

**Figure 1:**
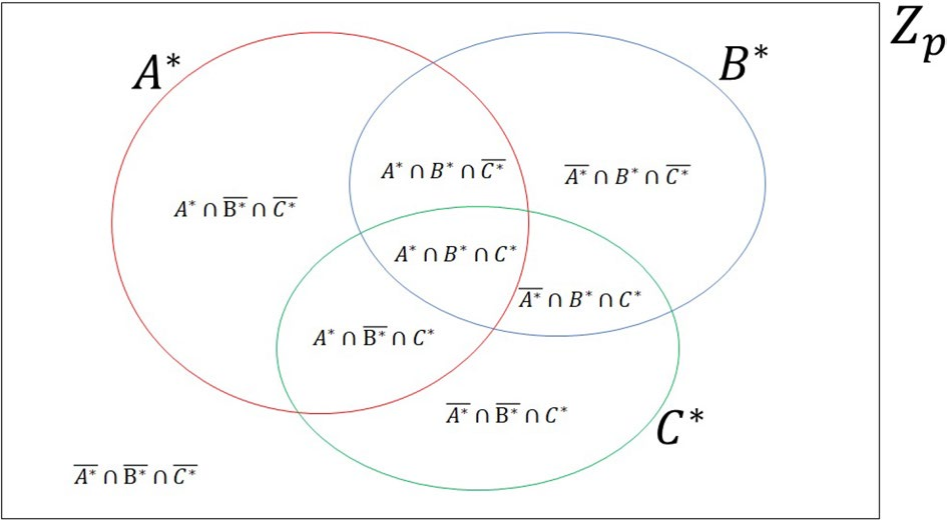
The relationships among $Z_{p}$, $A^{*}$, $B^{*}$ and $C^{*}$.

Furthermore, the relationships among $A^{*}$, $B^{*}$, $C^{*}$ and $Z_{p}$ can be illustrated by a Venn Diagram in Fig. 1, where red, blue, and green circles represent the sets $A^{*}$, $B^{*}$ and $C^{*}$, respectively, and $Z_{p}$ is the universal set. According to the Venn Diagram, we obtain the following equations:

5 $$\begin{aligned} \begin{aligned} |A^{*}\cap B^{*}|&=|A^{*}\cap B^{*}\cap {\overline{C^{*}}}|+|A^{*}\cap B^{*}\cap C^{*}|\\&=S_{110}+S_{111},\\ |A^{*}\cap C^{*}|&=|A^{*}\cap {\overline{B^{*}}}\cap C^{*}|+|A^{*}\cap B^{*}\cap C^{*}|\\&=S_{101}+S_{111},\\ |B^{*}\cap C^{*}|&=|{\overline{A^{*}}}\cap B^{*}\cap C^{*}|+|A^{*}\cap B^{*}\cap C^{*}|\\&=S_{011}+S_{111},\\ |A^{*}\cup B^{*}|&=|Z_{p}|-|{\overline{A^{*}}}\cap {\overline{B^{*}}}\cap C^{*}|-|{\overline{A^{*}}}\cap {\overline{B^{*}}}\cap {\overline{C^{*}}}|\\&=p-S_{001}-S_{000},\\ |A^{*}\cup C^{*}|&=|Z_{p}|-|{\overline{A^{*}}}\cap B^{*}\cap {\overline{C^{*}}}|-|{\overline{A^{*}}}\cap {\overline{B^{*}}}\cap {\overline{C^{*}}}|\\&=p-S_{010}-S_{000},\\ |B^{*}\cup C^{*}|&=|Z_{p}|-|A^{*}\cap {\overline{B^{*}}}\cap {\overline{C^{*}}}|-|{\overline{A^{*}}}\cap {\overline{B^{*}}}\cap {\overline{C^{*}}}|\\&=p-S_{100}-S_{000}, \\ |A^{*} \cup B^{*}\cup C^{*}|&=|Z_{p}|-|{\overline{A^{*}}}\cap {\overline{B^{*}}}\cap {\overline{C^{*}}}|\\&=p-S_{000}. \end{aligned} \end{aligned}$$

According to Eqs. (3)–(5), TP will finally obtain

6 $$\begin{aligned} \begin{aligned} |A\cap B|&=S_{110}+S_{111},\\ |A\cap C|&=S_{101}+S_{111},\\ |B\cap C|&=S_{011}+S_{111},\\ |A\cap B \cap C|&=S_{111},\\ |A\cup B|&=p-S_{001}-S_{000},\\ |A\cup C|&=p-S_{010}-S_{000},\\ |B\cup C|&=p-S_{100}-S_{000}, \\ |A \cup B\cup C|&=p-S_{000}, \end{aligned} \end{aligned}$$

which are the correct results.

**Table 1:** The relationship between *i* and three parties’ operations.

$i\in Z_{p}$	Alice’s operations	Bob’s operations	Charlie’s operations	TP’s measurment results
$i\in {\overline{A^{*}}} \cap {\overline{B^{*}}} \cap {\overline{C^{*}}}$	*I*	*I*	*I*	$|\varphi _{000}\rangle$
$i \in A^{*} \cap {\overline{B^{*}}} \cap {\overline{C^{*}}}$	*U*	*I*	*I*	$|\varphi _{100}\rangle$
$i \in {\overline{A^{*}}} \cap B^{*} \cap {\overline{C^{*}}}$	*I*	*U*	*I*	$|\varphi _{010}\rangle$
$i \in {\overline{A^{*}}} \cap {\overline{B^{*}}} \cap C^{*}$	*I*	*I*	*U*	$|\varphi _{001}\rangle$
$i \in A^{*} \cap B^{*} \cap {\overline{C^{*}}}$	*U*	*U*	*I*	$|\varphi _{110}\rangle$
$i \in A^{*} \cap {\overline{B^{*}}} \cap C^{*}$	*U*	*I*	*U*	$|\varphi _{101}\rangle$
$i \in {\overline{A^{*}}} \cap B^{*}\cap C^{*}$	*I*	*U*	*U*	$|\varphi _{011}\rangle$
$i \in A^{*} \cap B^{*} \cap C^{*}$	*U*	*U*	*U*	$|\varphi _{111}\rangle$

#### Security

In this subsection, we move on to the analysis of our protocol’s security. Two kinds of attacks, outside and participant attacks, on our protocol will be considered. Outside attacks come from an outside eavesdropper, Eve. Participant attacks can be launched by TP or dishonest parties.

*Outside attacks* In our protocol, TP and three parties employ decoy particles to prevent eavesdropping, which is derived from the BB84 QKD protocol^17^. And it has been proven to be unconditionally secure^18^. As we know, BB84 protocol remains secure even if the quantum channel is noisy, and our protocol can thus work on the noisy channels as well. Any eavesdropping will be detected in (Step 5) or (Step 8). Concretely, since the decoy state is randomly chosen from the set $\{|0\rangle ,|1\rangle |+\rangle |-\rangle \}$, it is in the state $\rho =\frac{I}{2}$. For any trio ($G_{Ai},G_{Bi},G_{Ci}$) ($i=0,1,\ldots ,p-1$), we have $\rho _{Ai}=\rho _{Bi}=\rho _{Ci}=\frac{I}{2}=\rho$. Eve thus cannot distinguish these two states. Without loss of generality, the most general strategy for Eve is that she performs an operation $U_{E}$ which causes the encoding states to interact coherently with an auxiliary quantum system $|e\rangle$, which can be described as follows:

7 $$\begin{aligned} \begin{aligned} U_{E}|0\rangle |e\rangle&= \alpha |0\rangle \left| e_{00}\right\rangle + \beta |1\rangle \left| e_{01}\right\rangle , \\ U_{E}|1\rangle |e\rangle&= \gamma |0\rangle \left| e_{10}\right\rangle + \delta |1\rangle \left| e_{11}\right\rangle , \end{aligned} \end{aligned}$$

where $|\alpha |^{2}+|\beta |^{2}=1$ and $|\gamma |^{2}+|\delta |^{2}=1$. In what follows, we will show that in order to pass the detection, Eve’s ancillary state and the encoding state should be product states.

From Eq. (7), if the decoy state is $|0\rangle$ or $|1\rangle$ and Eve introduces no error in the eavesdropping check, the following condition should be satisfied:

8 $$\begin{aligned} \beta = \gamma = 0. \end{aligned}$$

If the decoy state is $|+\rangle$ or $|-\rangle$ and Eve introduces no error in the eavesdropping check, we should have

9 $$\begin{gathered} U_{E} | + \rangle |e\rangle \hfill \\ = \frac{1}{{\sqrt 2 }}\left( {\alpha |0\rangle \left| {e_{{00}} } \right\rangle + \beta |1\rangle \left| {e_{{01}} } \right\rangle + \gamma |0\rangle \left| {e_{{10}} } \right\rangle + \delta |1\rangle \left| {e_{{11}} } \right\rangle } \right) \hfill \\ = \frac{1}{2}\left( {| + \rangle \left( {\alpha \left| {e_{{00}} } \right\rangle + \beta \left| {e_{{01}} } \right\rangle + \gamma \left| {e_{{10}} } \right\rangle + \delta \left| {e_{{11}} } \right\rangle } \right)} \right) \hfill \\ + \frac{1}{2}\left( {| - \rangle \left( {\alpha \left| {e_{{00}} } \right\rangle - \beta \left| {e_{{01}} } \right\rangle + \gamma \left| {e_{{10}} } \right\rangle - \delta \left| {e_{{11}} } \right\rangle } \right)} \right) \hfill \\ = \frac{1}{2}\left( {| + \rangle \left( {\alpha \left| {e_{{00}} } \right\rangle + \beta \left| {e_{{01}} } \right\rangle + \gamma \left| {e_{{10}} } \right\rangle + \delta \left| {e_{{11}} } \right\rangle } \right)} \right), \hfill \\ \end{gathered}$$

or

10 $$\begin{aligned} & U_{E} | - \rangle |e\rangle \\ & = \frac{1}{{\sqrt 2 }}\left( {\alpha |0\rangle \left| {e_{{00}} } \right\rangle + \beta |1\rangle \left| {e_{{01}} } \right\rangle - \gamma |0\rangle \left| {e_{{10}} } \right\rangle - \delta |1\rangle \left| {e_{{11}} } \right\rangle } \right) \\ & = \frac{1}{2}\left( {| + \rangle \left( {\alpha \left| {e_{{00}} } \right\rangle + \beta \left| {e_{{01}} } \right\rangle - \gamma \left| {e_{{10}} } \right\rangle - \delta \left| {e_{{11}} } \right\rangle } \right)} \right) \\ & + \frac{1}{2}\left( {| - \rangle \left( {\alpha \left| {e_{{00}} } \right\rangle - \beta \left| {e_{{01}} } \right\rangle - \gamma \left| {e_{{10}} } \right\rangle + \delta \left| {e_{{11}} } \right\rangle } \right)} \right) \\ & = \frac{1}{2}\left( {| - \rangle \left( {\alpha \left| {e_{{00}} } \right\rangle - \beta \left| {e_{{01}} } \right\rangle - \gamma \left| {e_{{10}} } \right\rangle + \delta \left| {e_{{11}} } \right\rangle } \right)} \right).{\text{ }} \\ \end{aligned}$$

Namely, the following equations

11 $$\begin{aligned} \begin{aligned} \alpha \left| e_{00}\right\rangle - \beta \left| e_{01}\right\rangle + \gamma \left| e_{10}\right\rangle - \delta \left| e_{11}\right\rangle&= 0, \\ \alpha \left| e_{00}\right\rangle + \beta \left| e_{01}\right\rangle - \gamma \left| e_{10}\right\rangle - \delta \left| e_{11}\right\rangle&= 0, \end{aligned} \end{aligned}$$

should hold, with 0 denoting a column zero vector. Depending on Eqs. (8) and (11), we can deduce that

12 $$\begin{aligned} \begin{aligned} \alpha =&\delta =1, \\ \beta =&\gamma =0,\\ |e_{00}\rangle&=|e_{11}\rangle . \end{aligned} \end{aligned}$$

Finally, we have

13 $$\begin{aligned} \begin{aligned} U_{E}|0\rangle |e\rangle&=|0\rangle \left| e_{00}\right\rangle , \\ U_{E}|1\rangle |e\rangle&=|1\rangle \left| e_{00}\right\rangle , \\ U_{E}|+\rangle |e\rangle&=|+\rangle \left| e_{00}\right\rangle , \\ U_{E}|-\rangle |e\rangle&=|-\rangle \left| e_{00}\right\rangle . \end{aligned} \end{aligned}$$

That is to say, Eve introduces no error in the eavesdropping only when her ancillary state and the encoding states are product states. Eve therefore cannot obtain useful information without being detected.

Note that our protocol involves two-way quantum transmission that may incur Trojan horse attacks^19,20^. We do not even need the photon number splitter and the optical wavelength filter devices^21,22^ to detect such an attack because the attacker knows nothing about three parties’ share key *k* that are employed to encrypt their private information.

*Dishonest parties’ attacks* Note that TP cannot collude with these dishonest parties. Suppose that Alice and Bob are the dishonest parties who intend to learn about Charlie’s set *C*. After they remove their respective decoy particles and do nothing on their particles, they may try to figure out what operations Charlie has perform on his particles to obtain $(z_{0},z_{1},\ldots ,z_{p-1})$. If Alice and Bob can get $(z_{0},z_{1},\ldots ,z_{p-1})$, they can easily steal Charlie’s private information because they share the same key *k*. Let’s consider the *i*-th trio of GHZ state. If $i\in C^{*}$, Charlie performs $U=ZX$ on the particle $G_{Ci}$ and then the state of $(G_{Ai},G_{Bi},G_{Ci})$ turns into $\left| \varphi _{001}\right\rangle _{G_{Ai}G_{Bi}G_{Ci}} =\frac{1}{\sqrt{2}}(|110\rangle _{G_{Ai}G_{Bi}G_{Ci}}-|001\rangle _{G_{Ai}G_{Bi}G_{Ci}})$; otherwise, the the state of $(G_{Ai},G_{Bi},G_{Ci})$ remains $\left| \varphi _{000}\right\rangle _{G_{Ai}G_{Bi}G_{Ci}}=\frac{1}{\sqrt{2}}(|000\rangle _{G_{Ai}G_{Bi}G_{Ci}}+|111\rangle _{G_{Ai}G_{Bi}G_{Ci}})$. In both case, $\rho _{G_{Ai}G_{Bi}}= \frac{1}{\sqrt{2}}(|00\rangle _{G_{Ai}G_{Bi}}\langle 00|+|11\rangle _{G_{Ai}G_{Bi}}\langle 11|)$, which means Alice and Bob cannot extract any private information from partial qubits of GHZ states. Thus, this attack by Alice and Bob is also invalid to our protocol.

*TP’s attacks* In our protocol, TP is assumed to be semi-honest, which means he will loyally execute the protocol, and he may use all the intermediate results to derive the other parties’ private information. However, he cannot collude with other dishonest parties.

Clearly, in 3.1, TP is able to derive $(x_{0},x_{1},\ldots ,x_{p-1})$, $(y_{0},y_{1},\ldots ,y_{p-1})$ and $(z_{0},z_{1},\ldots ,z_{p-1})$ according to the original GHZ states and the measurement results in the GHZ basis. Namely, TP knows what operations Alice, Bob and Charlie have done on their particles. Even though TP can then deduce whether or not $i \in A^{*}$ ($i \in B^{*}$,$i \in C^{*}$), he can still not learn any information about the elements in *A* (*B*, *C*). For example, suppose TP knows that $i \in A^{*}$ (i.e. $i = ka_{j} (\bmod \ p) \in A^{*}$), he knows nothing about $a_{j}\in A$ because he does not have the secret key *k*, whose security is guaranteed by Quantum Key Agreement. Hence, TP cannot steal three parties’ private information.

### Influence of Markovian Noise on the Protocol

In this section, assuming that the quantum state generator, quantum memories, and measurement devices in our protocol are perfect, we analyze the influence of six typical sorts of Markovian noise on our protocol. The effect of quantum noise on a tripartite quantum state $\rho _{123}$ can be characterized as follows:

14 $$\begin{aligned} \rho ^{\prime }_{123}=\sum _{i,j, k} K_{1}^{(i)} \otimes K_{2}^{(j)} \otimes K_{3}^{(k)} \rho _{123} K_{1}^{(i)\dagger } \otimes K_{2}^{(j)\dagger } \otimes K_{3}^{(k)\dagger }, \end{aligned}$$

where $\{K^{i}\}$ are Kraus operators characterizing quantum noise^23^ .

#### Flip channels

The flip channels have the following Kraus operators^23^

15 $$\begin{aligned} K^{(0)}=\sqrt{1-q} I, K^{(1)}=\sqrt{q} \sigma _{i}, \end{aligned}$$

where $i=1,2,3$ represents the bit flip ($\sigma _{1}=|0\rangle \langle 1|+|1\rangle \langle 0|$) , bit-phase flip ($\sigma _{2}=i(|1\rangle \langle 0|-|0\rangle \langle 1|)$) and phase flip ($\sigma _{3}=|0\rangle \langle 0|-|1\rangle \langle 1|$) channels, respectively, and $q\in [0,1]$ denotes the noise strength.

Suppose that the channel between TP and Alice, the channel between TP and Bob, and the channel between TP and Charlie are the same. We first consider the bit flip channel. For a GHZ state $\rho _{ABC}=|\varphi _{000}\rangle _{ABC}\langle \varphi _{000}|$ used for computation, after three particles arrived at Alice, Bob, and Charlie, respectively, the state of this tripartite system ABC becomes

16 $$\begin{aligned} \rho _{ABC}^{\prime }=\sum _{i,j, k=0}^{1} K_{A}^{(i)} \otimes K_{B}^{(j)} \otimes K_{C}^{(k)} \rho _{ABC} K_{A}^{(i)\dagger } \otimes K_{B}^{(j)\dagger } \otimes K_{C}^{(k)\dagger }, \end{aligned}$$

where $$K_{s}^{(0)}=\sqrt{1-q} I, K_{s}^{(1)}=\sqrt{q} \sigma _{1}$$ ($$s \in \{A,B,C\}$$).

Later, Alice, Bob, and Charlie perform unitary operations $U_{A}$, $U_{B}$ and $U_{C}$ on particle A, B, C, respectively, as described in (Step 6) of the proposed protocol, with $U_{A}$, $U_{B}$, $U_{C}$ $\in \{I, U=ZX\}$. We denote $U_{ABC}=U_{A}\otimes U_{B} \otimes U_{C}$, the state after Alice’s, Bob’s, and Charlie’s operations turns into

17 $$\begin{aligned} \rho _{ABC}^{\prime \prime }= U_{ABC}\rho _{ABC}^{\prime }U_{ABC}^{\dagger }. \end{aligned}$$

When TP receives these three particles from Alice, Bob, Charlie, the state of this system ABC is

18 $$\begin{aligned} \rho _{ABC}^{\prime \prime \prime }=\sum _{i,j, k=0}^{1} K_{A}^{(i)} \otimes K_{B}^{(j)} \otimes K_{C}^{(k)} \rho _{ABC}^{\prime \prime } K_{A}^{(i)\dagger } \otimes K_{B}^{(j)\dagger } \otimes K_{C}^{(k)\dagger }. \end{aligned}$$

When TP measures the system ABC in the GHZ basis, he expects to obtain the measure result $U_{ABC}\rho _{ABC}U_{ABC}^{\dagger }$, through which he can compute the cardinalities of intersections and unions, as described in 3.1 of our protocol. In this case, TP succeeds in the computation. We found that for all possible choices of $U_{ABC}$, the success probability is

19 $$\begin{aligned} P_\text {suc}^{\text {BF}}=1-6 q+18 q^2-24 q^3+12 q^4. \end{aligned}$$

Similarly, for bit-phase and phase channels, the success probabilities are

20 $$\begin{aligned} P_\text {suc}^{\text {BPF}}=(1-2 q+2 q^2)^3, \end{aligned}$$

and

21 $$\begin{aligned} P_\text {suc}^{\text {PF}}=1-6 q+30 q^2-80 q^3+120 q^4-96 q^5+32 q^6, \end{aligned}$$

respectively.

**Figure 2:**
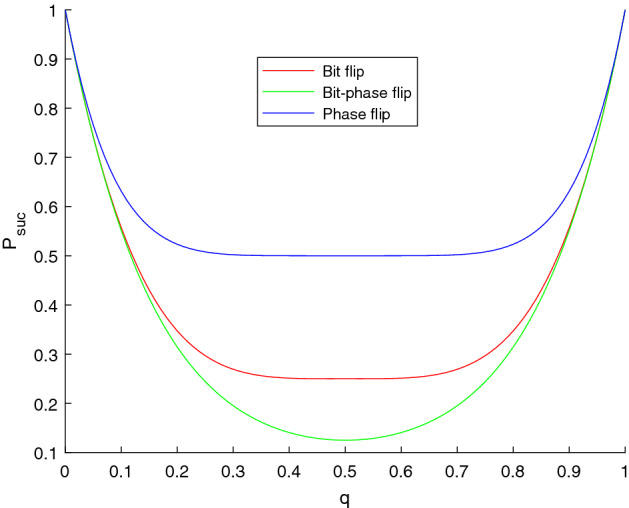
The variations of the success probabilities $P_{suc}$ using three kinds of flip channel with noise strength q.

The variation of these three success probabilities for flip channels are depicted in Fig. 2.

#### Depolarizing

The depolarizing channel can be characterized by the following Kraus operators^23^

22 $$\begin{aligned} &K^{(1)}=\sqrt{1-\frac{3}{4} q }I,&K^{(2)}=\frac{\sqrt{q}}{2} \sigma _{1},\\&K^{(3)}=\frac{\sqrt{q}}{2} \sigma _{2},&K^{(4)}=\frac{\sqrt{q}}{2} \sigma _{3}, \end{aligned}$$

where $\sigma _{1}=|0\rangle \langle 1|+|1\rangle \langle 0|$, $\sigma _{2}=i(|1\rangle \langle 0|-|0\rangle \langle 1|)$, $\sigma _{3}=|0\rangle \langle 0|-|1\rangle \langle 1|$, and $q\in [0,1]$ is the noise strength.

Suppose that the channel between TP and Alice, the channel between TP and Bob, and the channel between TP and Charlie are the same. Using the similar method in Flip channels analysis, the success probability of TP obtaining the correct measurement result is

23 $$\begin{aligned} P_\text {suc}^{\text {Dep}}=\frac{1}{8} (8-36 q+78 q^2-92 q^3+63 q^4-24 q^5+4 q^6), \end{aligned}$$

for all different choices of $U_{ABC}$.

#### Amplitude damping

The amplitude damping channel is used for the description of energy dissipation, which contains the Kraus operators^23^ as follows

24 $$\begin{aligned} K^{(1)}=\left[ \begin{array}{cc} {1} &{} {0} \\ {0} &{} {\sqrt{1-q}} \end{array}\right] , K^{(2)}=\left[ \begin{array}{cc} {0} &{} {\sqrt{q}} \\ {0} &{} {0} \end{array}\right] , \end{aligned}$$

where $q\in [0,1]$ is the noise strength.

Suppose that the channel between TP and Alice, the channel between TP and Bob and the channel between TP and Charlie are the same. Using the similar method in Flip channels analysis, the success probability of TP obtaining the correct measurement result is

25 $$\begin{aligned} \begin{aligned} P_\text {suc}^{\text {AD1}}=&\frac{1}{4} (4-12 q+21 q^2+(-22+8 \sqrt{1-q}) q^3\\&+(15-8\sqrt{1-q}) q^4-6 q^5+q^6), \end{aligned} \end{aligned}$$

for the cases where $U_{ABC}=I\otimes I \otimes I$ and $U_{ABC}=U\otimes U \otimes U$. For other cases, the success probability changes to

26 $$\begin{aligned} P_\text {suc}^{\text {AD2}}=-\frac{1}{4} (-1+q) (4-8 q+9 q^2+(-5+4 \sqrt{1-q}) q^3+q^4). \end{aligned}$$

#### Phase damping

The phase damping channel is characterized by the Kraus operators^23^ as follows

27 $$\begin{aligned} K^{(1)}=\left[ \begin{array}{cc} {1} &{} {0} \\ {0} &{} {\sqrt{1-q}} \end{array}\right] , K^{(2)}=\left[ \begin{array}{cc} {0} &{} {0} \\ {0} &{} {\sqrt{q}} \end{array}\right] , \end{aligned}$$

where $q\in [0,1]$ is the noise strength.

Suppose that the channel between TP and Alice, the channel between TP and Bob, and the channel between TP and Charlie are the same. Using the similar method in Flip channels analysis, the success probability of TP obtaining the correct measurement result is

28 $$\begin{aligned} P_\text {suc}^{\text {PD}}=\frac{1}{2} (2-3 q+3 q^2-q^3), \end{aligned}$$

for all sorts of choices of $U_{ABC}$.

**Figure 3:**
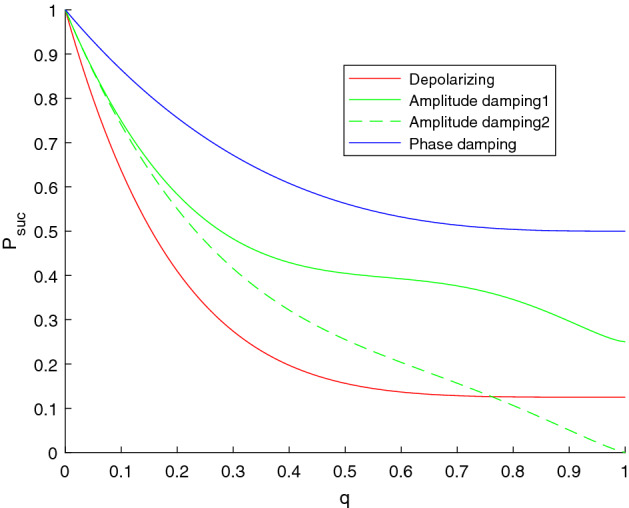
The variations of the success probabilities $P_{suc}$ on the depolarizing, amplitude damping, and phase damping channels with noise strength q.

The variations of the success probabilities of depolarizing, amplitude damping and phase damping channels are depicted in Fig. 3.

## Discussion and Conclusions

We presented a three-party protocol to compute the cardinalities of the intersection and the union between any two sets and among three sets with the help a semi-honest party TP. The security analysis showed that our protocol can resist some well-known quantum attacks. In addition, we analyzed the influence of six typical sorts of Markovian noise on the success probabilities of a GHZ state used for computation. The analysis showed that among three kinds of flip channels, the bit-phase flip channel affects our protocol most. It is interesting to see that on the amplitude damping channel, the success probabilities of the cases where $U_{ABC}=I \otimes I \otimes I$ and $U_{ABC}=U \otimes U \otimes U$ are the same, but for other cases, they share another success probabilities.

In practice, quantum error correction codes are usually employed to protect quantum states from errors induced by noise. There is also research on designing robust quantum cryptographic protocols based on some specific quantum states over special noisy channels (e.g. collective noise channels)^16^. Note that imperfect devices, such as quantum state generators and measurement devices, may also affect the robust of quantum cryptographic protocol, we will further conduct research on these topics in the future.
